# The Effect of the “Triple-Layer Medical Security” Policy on the Vulnerability as Expected Poverty of Rural Households: Evidence from Yunnan Province, China

**DOI:** 10.3390/ijerph191912936

**Published:** 2022-10-10

**Authors:** Jingjing Zhou, Yaoyu Zhang, Yong Sha, Jianfang Zhou, Hang Ren, Xin Shen, Hui Xu

**Affiliations:** 1 School of Sociology and Population Sciences, Nanjing University of Posts and Telecommunications, Nanjing 210023, China; 2 Institute of Population Studies, Nanjing University of Posts and Telecommunications, Nanjing 210042, China

**Keywords:** triple-layer medical security (TMS), vulnerability as expected poverty (VEP), registered poor households, Yunnan province, China

## Abstract

China launched the “critical battle against poverty” nationwide in 2012. As its main battlefield, Yunnan province promulgated the “triple medical security” (TMS) policy in 2017. This study, based on the pooled cross-section database of 2015–2020 of registered poor households in Yunnan province, employed the logit model to examine the effect of TMS on the vulnerability as expected poverty (VEP) of these households. It found that increasing the reimbursement rates for overall medical expenses and inpatient expenses and decreasing the proportion of out-of-pocket medical payment to income reduced the VEP; increases in the number of sick people in the family increased its VEP, and although the increase in the reimbursement rate for overall medical expenses or for inpatient expenses partially offset the VEP caused by the increase in the number of chronically ill people in the family, the VEP caused by the increase in the number of critically ill people would increase in the short term with the increase in the reimbursement rate for overall medical expenses or for inpatient expenses. The findings help improve policies concerning the medical security and health of the rural poor population, providing theoretical reference and practical guidance for future research.

## 1. Introduction

In September 2015, the United Nations General Assembly adopted the 2015–2030 Agenda for Sustainable Development with 17 Sustainable Development Goals at its core on the basis of the Millennium Development Goals. Poverty eradication as the primary goal has always been a concern of governments around the world [1,2,3]. In 2014, the Chinese government began to implement a targeted poverty alleviation strategy nationwide. Different from the previous anti-poverty measures, the targeted anti-poverty strategy emphasized precision in identification of poverty and in implementation of aid measures to ensure the accuracy of poverty alleviation effects [4,5,6]. In February 2021, 98.99 million rural poor people were lifted out of poverty, as were 832 poor counties and 128,000 poor villages, marking that China’s regional poverty, by and large, was addressed [7].

In China’s poverty alleviation efforts, disease is the most prominent poverty-causing factor for the rural population in China. By the end of 2019, there were 980,000 registered poor households (2.66 million people) that had not been lifted out of poverty in China, of which 375,000 households (968,000 people) were impoverished or returned to poverty due to illness, accounting for 38.4%. This rate remained at about 40% in 2016–2019 [8]. Moreover, the COVID-19 pandemic that began in late 2019 has increased health risks [9]. Globally, the COVID-19 pandemic has thrown into disorder health services and overwhelmed health systems across the world. Even before the pandemic, 500 million people had been pushed into (or further into) extreme poverty by paying for medical care. The number by some estimates is much higher now [10]. Therefore, solving the problem of poverty and return to poverty caused by illness is not only the focus and difficulty of poverty governance in China in the future, but it is also a global challenge.

In its implementation of the targeted anti-poverty strategy, China finds that health poverty alleviation plays an important role. As its core is the “triple-layer medical security” policy (TMS). TMS encompasses basic medical insurance, critical illness insurance and medical assistance. It ensures the fairness of the medical security system by tilting towards the registered poor population with preferential reimbursement of medical expenses for basic medical care and critical illness and increased medical assistance. Compared with the general public, the registered poor population enjoys higher rates of reimbursement for in-patient, out-patient and emergency medical services, lower threshold to reimbursement and higher cap of reimbursement amount for critical illness treatment. For those still struggling with personal out-of-pocket payment after reimbursement, assistance is provided according to the actual situation. TMS covers the vulnerable groups affected by the disease risk to the greatest extent [11,12].

In addition, China has ushered in a post-poverty alleviation era characterized by transitional secondary poverty and relative poverty [13]. Pre-measurement of the probability of poverty is the key to cutting off the causal link between disease and poverty and to reducing the number of rural people falling into disease-induced poverty. Poverty vulnerability overcomes the static and ex post facto nature of conventional poverty measurement and can predict the probability of poverty from a forward-looking perspective [14]. This study, therefore, investigated the health poverty alleviation practice in Yunnan province, an important main battlefield for China’s poverty alleviation. Using the province’s data on registered poor households provided by the Poverty Alleviation Office of Yunnan Province and the province’s medical reimbursement data provided by the Medical Insurance Bureau of Yunnan Province, we built a high-quality pooled cross-section database of the poor population from 2015 to 2020, and analyzed the database to verify the policy effect of TMS on the vulnerability to poverty (VP)—the probability of remaining poor or falling deeper into poverty (probability of poverty, collectively)—measured by vulnerability as expected poverty (VEP) of the households with an view to providing forward-looking thinking and theoretical support for the governance practice of poverty alleviation through healthcare in China and beyond.

## 2. Literature Review and Research Hypotheses

### 2.1. Literature Review

#### 2.1.1. VP and VEP

Conventional poverty measurement statically quantifies the welfare level of families or individuals at a certain point in time and cannot predict future welfare status and the risks associated with it. Policies based on pre-existing poverty may be ineffective for those on the edge of poverty [15]. In 2000, the concept of VP was first proposed by the World Bank to indicate the probability of welfare decline due to various risk shocks. It described vulnerability as the exposure to various risk shocks of families or individuals and the probability of their income loss or quality of life falling below the poverty line due to the risk shocks [16]. Current literature has not formed a uniform concept or definition of “VP”. For example, Pritchett et al. (2000) thought of it as the probability of a household becoming poor in a certain year over the next several years [17]. Similarly, Chaudhuri et al. (2002) defined a household’s VP at time T as the probability of it becoming poor at T + 1 [18]. A small number of studies have made different definitions. Glewwe and Hall (1998) believed that vulnerability is a dynamic concept, encompassing a series of consequences caused by macroeconomic shocks, in which market-induced vulnerability can be measured by changes in household consumption [19].

As seen from above, different definitions merit different measurements. For ease of operation and measurement, VP is measured by the following three quantitative approaches, namely VEP, VEU (vulnerability as expected utility) and VER (vulnerability as exposure to risk). VEP is viewed as the likelihood of a household becoming poor in the future [18]. VEU is seen as the gap between the utility from a certain level of certainty-equivalent consumption (usually defined as the poverty line) and the expected utility of consumption. A household is considered vulnerable if the expected utility of consumption is below the utility from some certainty-equivalent consumption level [20]. VER is seen as a rapid decline in consumption levels when farmers are exposed to risk shocks [17]. Among the three viewpoints, both VEP and VEU are ex ante measures of welfare losses that households may suffer from risks, with the difference in the definition of “welfare loss”. Scholars in support of VEP define “welfare loss” as the gap between the worsened condition in the future and a certain welfare target, while those who agree with VEU understand “welfare loss” as a decrease in utility. Unlike VEP and VEU, VER is an ex post concept which, instead of directly estimating total vulnerability, measures vulnerability by estimating the sensitivity of consumption to income changes caused by risk shocks. The greater the changes in income caused by risk shocks, the higher the vulnerability of consumption to income risks [14,15].

#### 2.1.2. Research on Disease and VP

Relevant scholars have conducted a lot of theoretical and empirical research on the relationship between health and poverty, and most of them believe that poor health is an important contributor to VP [21]. Some scholars have opined that VP should include not only unstable income, but also risks related to health, risks from social exclusion, etc., and these risks will have an important impact on the welfare status of families [22,23,24]. Others have directly measured the linkage between health and VP, arguing that VP is closely related to individual health status. For every 10% decrease in individual health level, VP will increase by 6% [25].

On the basis of revealing the relationship between health and VP, there are studies that focus on the impact of disease on the VP in the ex ante sense, i.e., VEP. Chronic and critical illnesses have become an important cause for the impoverishment of families with the sick [26,27]. The main reason is that diseases (chronic or critical diseases) are difficult to eradicate and easy to recur, and that the rural poor are more vulnerable to poor health due to their economically disadvantaged status [28], which will reduce their family income and increase treatment costs, causing heavy economic burdens [29]. Compared with the general population, the rural poor population is more exposed to the direct economic burden caused by disease [30]. Sickness, disability and death result in a reduction in effective working time and capacity, causing economic losses to families and individuals [31]. In low- and middle-income countries, the medical expenses and the cost of lost work brought about by treatment for such diseases as hypertension, coronary heart disease and stroke present greater direct and indirect economic burdens [32,33] on social groups in economically disadvantaged positions, reducing their quality of life.

It can be seen that diseases cause two major economic burdens of medical costs and reduced labor supply to ordinary families, which will not only lead to a decline in family income, but also lead to a vicious circle of poverty. A substantial decline in living standards pushes these families into more severe poverty [34]. Whether in China or around the world, long-course or recurring diseases, especially critical ones, are still an important cause of poverty or poverty reentry.

#### 2.1.3. Research on Medical Security System and VP

Many scholars have carried out research on the poverty reduction performance of medical security policies. Some scholars have verified the direct or potential role of anti-poverty projects in promoting health through RCTs (Randomized Controlled Trials), among other trials, proving the sustainable positive effects and cost-effectiveness of these projects [35,36]. Some have used the catastrophic health expenditure (CHE) and the impoverishment by medical expense (IME) methods recommended by the WHO to measure the economic burden of diseases on the poor, followed by the use of propensity score matching, regression discontinuity design (RDD), double difference and other quasi-experimental methods to verify the anti-poverty effect of medical security or insurance, the main measures of poverty alleviation through healthcare [37,38,39,40].

In addition, a handful of studies have further explored the action of medical insurance on poverty reduction. Medical insurance can increase labor supply by improving health status, reduce precautionary savings to increase productivity and human capital investment, and reduce the burden of medical expenditures, thereby achieving a poverty reduction effect [39,41]. Participants in medical insurance report a sound policy effect in addressing poverty or poverty reentry due to illness as increased coverage of critical illness insurance can significantly reduce the VEP [42,43]. Public health insurance encourages the insured to utilize medical services and significantly reduces the risk of CHE, helping to prevent the impoverishment of families hit by disease [44]. In the United States, medical insurance has significantly reduced the out-of-pocket medical expenditure of the elderly over 65 years old by 33%, effectively reducing their medical burden, and thus seeing a significant poverty reduction effect [45,46]. In Ghana, the health insurance system has significantly lowered households’ out-of-pocket healthcare expenditures by approximately 86% and poverty rate by about 7.5% [47]. Some scholars, however, hold that no anti-poverty effect is derived from medical insurance. They argue that the compensation offered by insurance fails to alleviate the poverty of families with sick members but increases their high-risk and catastrophic expenditures [48]. The putative explanation is that, on the one hand, the level of insurance is low, the targeting mechanism is poor, and the effect takes a long period to present itself [49]; on the other hand, medical insurance incentivizes people to seek treatment and higher-level care when falling ill [50].

### 2.2. Research Hypotheses

As suggested in the literature, existing studies mainly use micro-sampling data to test the anti-poverty effect of medical insurance or of certain medical insurance schemes [51]. However, they are prone to small sample bias and the omission of unobservable variables that also causes bias in the cross-regional analysis, thereby affecting the reliability of conclusions to a certain extent [52]. Moreover, compared with a single scheme, multi-layer medical security delivers a more significant effect in poverty reduction [53]. Since few scholars have paid attention to the empirical research of multi-layer medical security policies [54], this study used more comprehensive multi-period data to probe the effect of a TMS policy on the VEP of rural registered poor households.

Based on the above research, we hypothesized that the TMS policy reduces the VP of Chinese rural registered poor households and improves their resilience in the face of poverty risks brought on by health shocks. Specifically, the following hypotheses were proposed:

**Hypothesis** **1.** *The higher the inpatient reimbursement rate, the lower the VEP of rural registered poor households.*

**Hypothesis** **2.** *The higher the overall reimbursement rate, the lower the VP of rural registered poor households.*

**Hypothesis** **3.** *The higher out-of-pocket medical expenses as a proportion to total income, the higher the VP of rural registered poor households is.*

**Hypothesis** **4.** *The more the chronically ill members in the family, the higher its VP is.*

**Hypothesis** **5.** *The more the critically ill members in the family, the higher its VP is.*

## 3. Materials and Methods

### 3.1. Study Setting and TMS Policy

**Figure 1:**
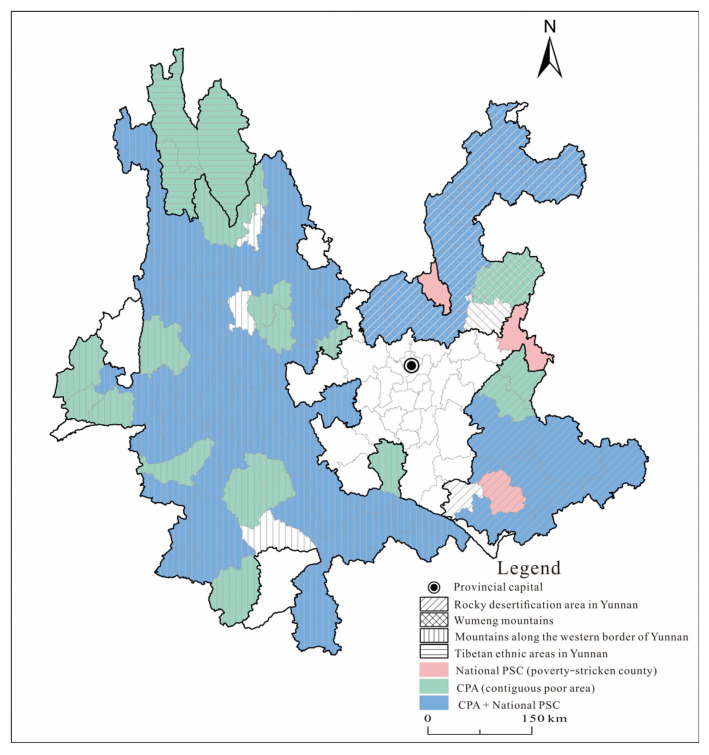
Distribution of CDAs and poor counties in Yunnan in 2020.

**Figure 2:**
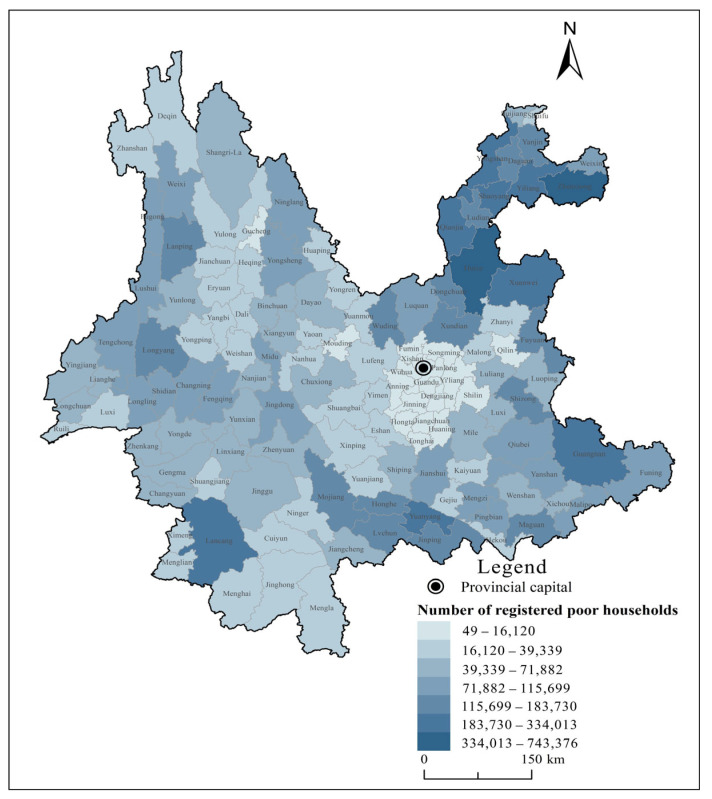
County-level distribution of registered poor households in Yunnan in 2020.

Yunnan province was chosen as the research region for two reasons. Geographically, it is located in the southwestern border of China, with a land area of 394,000 square kilometers, of which more than 94% are mountainous and semi-mountainous areas. It borders with Myanmar, Laos, and Vietnam along the line of 4060 km. With a large population that is broadly and deeply poor, it is a “four-in-one” province, namely a frontier, multi-ethnic, mountainous, and poor [55]. In terms of poverty level, by calculation using the national rural poverty line at an annual per capita income of CNY 2300 (at constant price in 2010) derived from the data released by the National Bureau of Rural Development, in 2013 a total of 82.49 million poor people were identified across the country, and the poverty-stricken population in Yunnan province was 6.61 million, representing 8.01% of the poor population nationwide [56]. The count of poor people in Yunnan province was second only to that in Guizhou province, and the poverty incidence rate in Yunnan province was 17.8%, ranking fifth in the country. In 2011, the Outline of Poverty Alleviation and Development in China’s Rural Areas (2011–2020) designated 14 contiguous destitute areas (CDA) as the main battlefield of poverty alleviation. They basically cover the vast majority of deeply impoverished population in poverty-stricken areas in the country [57]. Among the 14 CDAs in the country, Yunnan province accounted for four with 91 counties, ranking first in the country (see Figure 1 for details) [58]. According to the Poverty Alleviation Plan of Yunnan Province (2016–2020), at the end of 2015, Yunnan province had 4.71 million registered poor people, 88 poverty-stricken counties, and 4277 poverty-stricken villages, with a poverty incidence rate of 12.7%. Figure 2 presents the number of registered poor households in each county in Yunnan province as of 2020. As can be seen from the figure, the poverty-stricken population in Yunnan province was distributed basically where poverty-stricken areas were distributed, and a few counties tend to see a high concentration rate, such as Zhenxiong and Huize [59].

With the deepening of poverty alleviation in Yunnan, the poor population has been shrinking year by year, but among them the rate of return to poverty due to illness has risen from 10.10% in 2014, 11.73% in 2015 to 18.91% in 2016 [60]. This entails an extremely arduous task of health poverty alleviation in Yunnan because Yunnan’s CDAs are mostly located in remote areas with adverse natural conditions and high incidence of endemic diseases, making it difficult for conventional measures of health poverty alleviation to fundamentally reduce the risk of poverty caused by health shocks [61]. In 2017, the province formulated the Thirty Measures for Poverty Alleviation through Healthcare in Yunnan Province in light of the actual situation. The document proposed the implementation of the TMS policy from 2017 onwards. Since the implementation, the actual reimbursement rate of compliant medical expenses for registered poor patients in Yunnan increased from 61.15% in 2016 to 89.45% by the end of 2020, and the per capita out-of-pocket expenses dropped from CNY 2241.63 in 2016 to CNY 696.01 at the end of 2020. On top of that, such policies as “payment after treatment” and “one-stop reimbursement” have been fully realized at designated medical institutions at the county level of the province [62].

### 3.2. Data Collection

The data used herein was generated in three ways as below.

First, China’s national poverty alleviation information system, the most authoritative database operated by the State Council, went online in 2014. It contains the data of all registered poor individuals, households and villages across the country [63], including the family population, income, land management, living conditions, economic development of the administrative villages, etc. The total sample in this study consisted of more than 16 million poor households registered in 2013–2020, a study period with relatively complete data.

Second, Yunnan province has been committed to expanding the coverage of primary medical care, such as health cards and contracted medical services, to all poor households since 2015. However, large-scale campaigns of poverty alleviation through healthcare were carried out only after 2016, and the 30 Measures for Poverty Alleviation through Healthcare was not adopted until late 2017 [64]. In this study, data on medical payment and reimbursement of all the registered population were derived from the official system of Yunnan Provincial Medical Security Bureau, which was then organized into the “household + year” format and horizontally merged with the registration data to finally obtain 1,448,496 households, forming a pooled cross-section dataset.

Third, we adjusted the household’s per capita net income (net of productive expenditures) and poverty line represented by income for inflation using the annual Consumer Price Index (CPI) of each prefecture in Yunnan Province obtained from the previous statistical bulletins on economic and social development of each prefecture, thus ensuring the comparability of data over the years. In the fixed-base processing, the CPI data over the years was processed with 2014 as the base period, in which the 2014 CPI was assigned a value of 100, and then the income data was adjusted for inflation using the fixed-base CPI to obtain the comparable income data with 2014 as the base period [65]. The poverty lines represented by income in Yunnan province over the years were derived from official reports [66].

### 3.3. Variables

#### 3.3.1. Explained Variable

The explained variable herein is the probability of poverty among the registered poor households. Related research usually adopts the probability value converted to a different time period as the VEP threshold [67]. A common practice is to convert the 50% probability value of the household being poor into its equivalent in the next two years, i.e., the 29% probability value [68]. Therefore, this study chose 29% and 50% as the VEP threshold, respectively, to generate the outcome variables, and the model with the 50% threshold went through the robustness test. Specifically, with the 29% VEP threshold, the outcome variable was generated as probability of poverty 1, i.e., if VEP exceeds 29%, the rural household has the probability of poverty, which took the value of 1, or otherwise 0. With the 50% VEP threshold, the outcome variable was generated as probability of poverty 2, i.e., if VEP exceeds 50%, the rural household has the probability of poverty, which took the value of 1, or otherwise 0.

#### 3.3.2. Core Explanatory Variables

In order to test the linkage between the TMS policy and VP, four core variables were selected: (1) the inpatient reimbursement rate represented by a percent, denoting the annual compensation received by the registered poor household under severe health shocks relative to its inpatient expenses in the designated hospital [69]; (2) the overall reimbursement rate represented by a percent, denoting the compensation rate of annual medical expenses for all medical services including inpatient and outpatient treatment of the registered poor household in the designated hospital [70]; (3) the proportion of out-of-pocket expenses to income represented by a percent, denoting the out-of-pocket medical expenses paid by the registered poor household as a percentage of the total annual income of the year [71,72]; (4) further, the inpatient reimbursement rate or the overall reimbursement rate was multiplied by the number of chronically ill members in the family or the number of critically ill members in the family, respectively, to obtain the interaction terms, so as to better observe how medical reimbursement acts on the poverty of rural households [73].

#### 3.3.3. Control Variables

The following control variables were introduced: household population, characteristics of the household head, agricultural management status, location conditions and village-level economic characteristics (see Table 1).

### 3.4. Statistical Analysis

#### 3.4.1. Measurement of VP

As mentioned above, approaches developed in the literature to measure vulnerability include VEP [17], VEU [74] and VER [75]. The VEP approach proposed by Chaudhuri et al. (2002) is widely used in the research. It is a measure of the probability of the target individual or family becoming poor in the future [18]. The advantage of this approach is that VP can be estimated using cross-sectional data, overcoming the shortage of missing panel data. The VEP studied in this paper describes the probability of the registered poor household to remain poor or fall deeper into poverty (to be poor collectively) due to health shocks. Based on existing research, this study adopted three-step feasible generalized least squares (FGLS) to calculate the VEP defined as the likelihood of a household falling under the poverty line measured by its net per capita income [18,76,77].

First, we obtained the fitted values and squared residuals with the income equation. A multiple linear regression (MLR) model was built with the characteristics of the head of household, household features, health conditions of family members, and the community (village) as explanatory variables, and the household income per capita as the response variable, as shown in Equation (1), where ln*Y_i_* is the log of net per capita income, and $X_{ir}$ is a set of explanatory variables as explained above.

(1) $$\ln Y_{i}=\beta_{0}+\beta_{r}X_{ir}+\varepsilon$$

Second, we performed weighted regression on the log of net per capita income and squared residuals to cure heteroscedasticity. We obtained the values of FGLS estimators: $\hat{\beta }$ and $\hat{\theta }$, and then the predicted value and variance of the log of future net per capita income, as illustrated in Equations (2) and (3).

(2) $$\hat{E}\left[\ln Y_{h}\left|X_{h}\right.\right]=X_{h}\hat{\beta }$$

(3) $$\hat{V}\left[\ln Y_{h}\left|X_{h}\right.\right]=X_{h}\hat{\theta }$$

Lastly, we obtained the VEP by plugging the results obtained in the previous steps into Equation (4), where $VUL_{it}$ was the VEP of Household *i* in Year *t*, *Z* the poverty line, and Pr was the probability of a household being under the poverty line. The VEP was calculated over the period from 2014 to 2019, and the recognized poverty lines, measured by the per capita income per year, were CNY 2300 in 2014, CNY 2800 in 2015, CNY 2952 in 2016, CNY 3200 in 2017, CNY 3500 in 2018, CNY 3750 in 2019, and CNY 4000 in 2020.

(4) $$VUL_{it}=\mathrm{Pr}\left(Y_{i,t+1}\leq Z\right)$$

#### 3.4.2. Logit Model

If the explained variable is a discrete variable rather than a continuous one, such as whether or not to be poor, whether or not to be employed, whether or not to join a labor union, or whether or not to go abroad, then it takes the value of 1 or 0 to indicate Yes or No, which are called binary choices [78]. At this time, the distribution of y was a two-point distribution, as shown in the following Formula (5). $F\left(x,\beta \right)$ was a link function, which connected the explanatory variable and the explained variable. $\hat{y}$ was understood as the probability that y = 1 occurs (as shown in Equation (6)). Assuming $F\left(x,\beta \right)$ as the cumulative distribution function that conforms to the logistic distribution (as shown in Equation (7)), it was a Logit model, that is, a nonlinear model, and the maximum likelihood estimation method was used. It should be noted that in this nonlinear model, the estimator ${\hat{\beta }}_{MLE}$ was not a marginal effect, and for nonlinear models, the marginal effect itself is not constant, but varies with the explanatory variable [79]. However, Equations (8) and (9) were obtained based on ${\hat{\beta }}_{MLE}$, where $p\left(1-p\right)$ was called the odds ratio or relative risk. We suppose that to estimate the probability of poverty, y=1 means being poor, y=0 means not being poor, and if the odds ratio is 2, this means that the probability of being poor is twice that of not being poor. If the odds ratio of the variable of age is 1.2, it means that, holding other variables constant, for every 1-year increase in age, the probability of being poor increases by 20%. In this study, the explained variable (whether or not to be poor) was a 0–1 binary response variable. Drawing on existing research, this study used the logit model for estimation [80].

(5) $$\left\{\begin{array}{c} P\left(y=1\left|x\right.\right)=F\left(x,\beta \right)\\ P\left(y=0\left|x\right.\right)=1-F\left(x,\beta \right) \end{array}\right.$$

(6) $$E\left(y\left|x\right.\right)=1\cdot P\left(y=1\left|x\right.\right)+0\cdot P\left(y=1\left|x\right.\right)=P\left(y=1\left|x\right.\right)$$

(7) $$P\left(y=1\left|x\right.\right)=F\left(x,\beta \right)=\wedge \left(x^{\prime }\beta \right)=\frac{\exp\left(x^{\prime }\beta \right)}{1+\left(x^{\prime }\beta \right)}$$

(8) $$\frac{p}{1-p}=\exp\left(x^{\prime }\beta \right)$$

(9) $$\ln\left(\frac{p}{1-p}\right)=x^{\prime }\beta$$

## 4. Results

### 4.1. Descriptive Statistics

The variables herein are described statistically in Table 2.

If VEP exceeds 29%, 24% of the total sample has the probability of poverty; if VEP exceeds 50%, 12% of the total sample has the probability of poverty.

From the perspective of medical reimbursement, the inpatient reimbursement rate had a minimum value of 0, maximum value of 100, median of 90.00 and mean of 79.03, indicating that the overall inpatient reimbursement rate for rural registered poor households was relatively high, but a certain gap existed in the level of inpatient reimbursement between them; the medical reimbursement rate for all medical services including outpatient and hospitalization had a minimum value of 0.04, maximum value of 99.99, median of 78.89 and mean of 75.30, indicating a high rate of overall reimbursement and a small gap in this aspect among rural registered poor households; the proportion of out-of-pocket payment to income had a minimum value of 0.02, maximum value of 190.79, median of 6.63 and mean of 13.66, indicating a big gap in this aspect among the households.

With respect to family demographics, the number of the chronically ill and the number of the critically ill had a minimum value of 0 each, maximum values of 4 and 3 and means of 0.41 and 0.12, respectively, indicating that the proportion of chronically or critically ill people in the family was relatively small, but the proportion of chronically ill was higher than that of critically ill people in the family.

From the angle of household head’s characteristics, the head of the household had an average age of 50.82 years old and an education level with a minimum value of 1, maximum value of 6 and mean of 2.13, indicating that, among the household heads, the average education level was primary school and below, the highest education level was only junior high school and the overall education level was low. The number of migrant workers had a minimum value of 0, maximum value of 6 and mean of 0.55, indicating that most registered rural poor households chose to stay in the local area than go out to work.

In aspect of agricultural management status, the cultivated area had a minimum value of 0 mu, maximum value of 316.4 mu and mean 6.83 mu; the forest area had the minimum value of 0 mu, maximum value of 1200 mu and mean of 13.71 mu, indicating that the rural household’s forest area was generally larger than their cultivated area, and 43% of the farmers were members of specialized cooperatives.

In terms of location conditions, the availability of radio and TV channels had a minimum value of 0, maximum value of 1 and mean of 0.96; the distance from the rural main roads had a minimum value of 0 km, maximum value of 2000 km and mean of 1.23 km; the availability of asphalt/concrete roads entering other villages/towns had a minimum value of 0, maximum value of 1 and mean of 0.96; and the availability of passenger shuttle buses had a minimum value of 0, maximum value of 1 and mean of 0.75, suggesting that the vast majority of villages had access to radio and TV channels, hardened roads and regular passenger buses to other towns, though the distance from the main rural roads was relatively large.

As to village-level economic characteristics, the village-level collective economic income had a minimum value of CNY 0, maximum value of CNY 2.06 million and mean of CNY 700; the number of farmers’ specialized cooperatives had a minimum value of 0, maximum value of 267 and mean of 3.16, indicating relatively large economic gaps at the village level.

### 4.2. Modeling Results

Table 3 lays out the model estimation results. The first and third columns in Table 3 report the coefficients estimated by the logit model. In order to show the model estimation results more intuitively, the second and fourth columns report the odds ratio. It can be seen from the results that all variables withstood the significance test. From the perspective of household demographics and household head’s characteristics, the coefficients of the number of migrant workers, the household head’s education level and age were all negative, indicating that the more migrant workers, the higher the education level and the older the household head, the lower the VP; from the standpoint of agricultural management status, the coefficients of cultivated area, forest area and whether to have joined a farmers’ specialized cooperative were all negative, indicating that larger cultivated area and forest area owned by the family or the participation in farmers’ specialized cooperatives was conducive to reducing VP; from the perspective of location conditions, the coefficients of the availability of radio and television, hardened roads leading to the township and shuttle passenger buses were all negative, while the coefficient of the distance from rural main roads was positive, indicating that access to radio and television, hardened roads leading to the township and shuttle passenger buses significantly reduced the VP, and that the farther away from the main roads of the village, the higher the VP, suggesting that sound village-level infrastructure construction can significantly reduce the VP; from the perspective of the village’s economic characteristics, the coefficient of the village collective economic income was positive and the coefficient of participation in specialized cooperatives was negative, suggesting that higher collective economic income in the village led to higher VP, and that the more the membership in farmers’ specialized cooperatives in the family, the lower its VEP. Except for the village’s collective economic income, the directions of action of other variables on the VEP were basically in line with expectations. An increase in the village collective income aggravated poverty, implying that improvement shall be made in the organization of the collective economy and in the pattern of income distribution. The collective economy is an important part of the socialist economic organization, and rural specialized cooperatives are an important way to promote the development of the collective economy [81]. Rural specialized cooperatives that champion interest sharing share the risks of farmers’ economic investment to varying degrees. Although the collective income is considerable, there are still problems such as uneven distribution of benefits and large differences in the distribution of benefits. The distribution of equity in specialized rural cooperatives is so concentrated that most farmers, as retail investors, only hold a small part of the equity, resulting in less investment and less return, which cannot fundamentally eliminate poverty [82].

Regarding the key variables such as the reimbursement rate concerned in this study, both the inpatient reimbursement rate and the overall reimbursement rate were significantly negatively correlated with the VEP of rural families at the 1% confidence level, and the estimated coefficients were similar to each other, indicating that both had the effect of reducing poverty. Whether it is inpatient reimbursement for more serious health problems or the overall reimbursement (including outpatient, hospitalization and others), both had the effect of reducing poverty, and their effect of reducing poverty was similar. Correspondingly, the proportion of out-of-pocket payment to income had a significant positive effect, indicating that it aggravated poverty. From the viewpoint of the odds ratio, for every 1% increase in the inpatient reimbursement rate and the overall reimbursement rate, the odds of being poor decreased by 4%. By the same token, every 10% increase in the inpatient reimbursement rate and the overall reimbursement rate reduced the odds of being poor by 40%. It can be seen that increasing the medical reimbursement rate indeed effectively reduced and prevented poverty. For the out-of-pocket payment as a proportion to income, for every 1% increase, the odds of poverty increased by 0.4%. Similarly, when it increased by 10%, 20%, and 30%, the odds of poverty increased by 4%, 8% and 12%, respectively, indicating that the greater the burden of medical expenditure on the household, the greater the probability of poverty.

Regarding the number of chronically ill members, the number of critically ill members and their interaction terms, the estimated results show that the odds ratios of the number of the chronically ill in the two models were 1.254 and 1.263, respectively. In other words, for every additional patient with chronic illness in the family, the odds of poverty increased by as much as 25% compared to the odds of not being poor at 26%. Similarly, as suggested by the odds ratio of the number of the critically ill, for every additional patient with critical illness in the household, the probability of poverty increased by as much as 17% compared to the odds of not being poor at 12%. Moreover, according to the interaction terms constructed by multiplying the number of patients and the reimbursement rate, both the inpatient reimbursement rate × number of chronically ill patients and the overall reimbursement rate × number of chronically ill patients were significantly negatively correlated with the probability of poverty at the 1% confidence level, hinting that the risk of poverty brought about by the increase in the number of chronically ill people could be partially offset by the increase in the overall reimbursement rate or the inpatient reimbursement rate. On the contrary, both the inpatient reimbursement rate × number of the critically ill and the overall reimbursement rate × number of the critically ill were significantly positively correlated with the probability of poverty at the 1% confidence level, indicating that the exposure to poverty caused by the increase in the number of critically ill members in the family will heighten with the increase in the reimbursement rate for overall medical expenses or the reimbursement rate for inpatient expenses.

### 4.3. Robustness Test

We adopted the transformation of explained variables to test the estimated results’ robustness. We selected a 50% VEP threshold to generate the categorical variable: probability of poverty 2 (if VEP exceeds 50%) and used the logit model to verify it. Table 4 displays the model estimation results as below, where the first and third columns report the improved logit model estimation results, and the second and fourth columns report the improved odds ratios. At the 1% level, the inpatient reimbursement rate, the overall reimbursement rate, the inpatient reimbursement rate × number of chronically ill people, and the overall reimbursement rate × number of chronically ill people all significantly negatively affected rural families’ probability of poverty. At the 1% level, the numbers of the chronically ill and of the critically ill, the inpatient reimbursement rate × number of critically ill patients, and the overall reimbursement rate × number of critically ill patients all had a significant positive impact on rural families’ probability of poverty.

It can be seen from the results that there was no significant change in the sign and significance of the estimated coefficients of all variables, suggesting that there was no fundamental difference in the estimated results, and that the poverty prevention and reduction effects of the overall reimbursement rate or the inpatient reimbursement rate were still significant. The estimated results of this study were therefore robust.

## 5. Discussion

Although poverty alleviation in the “blood-transfusion” model can quickly reduce poverty in the short term, the effect is not good in the long run [83]. The Chinese government proposes that the top priority should be to consolidate the achievements of poverty alleviation. To that end, it extends the policy validity to ensure that those who have been lifted out of poverty will not return to poverty, and deepens poverty alleviation by rural revitalization, also known as the “blood-making” model of poverty alleviation [84]. In this context, we examined the effect of the TMS policy on the VP of registered poor households in Yunnan province. Five research questions were verified in this study: (1) Will the increase in the inpatient reimbursement rate reduce the VEP of rural registered poor households? (2) Will the increase in the overall reimbursement rate reduce the VP of rural registered poor households? (3) Will the increase in the proportion of out-of-pocket medical expenses to total income increase the VP of rural registered poor households? (4) Will the more chronically ill members in the family increase its VP? (5) Will the more critically ill members in the family increase its VP?

In this study, increases in the medical reimbursement rate could indeed effectively reduce and prevent poverty. Whether it is for inpatient reimbursement for more serious health problems or overall reimbursement (including outpatient, inpatient, and others), increases in the medical reimbursement rate could reduce the VEP of rural registered poor households. This finding supports Hypotheses 1 and 2 in this study and is also consistent with previous findings. Increased coverage of critical illness insurance significantly reduces VP [43]. Increasing the overall reimbursement rate is conducive to releasing poor households’ long-suppressed utilization of medical services, thereby improving physical health, bringing about an “enhancing effect” and “stabilizing effect” on income. Under the “enhancing effect”, the household’s bread-earners with better health obtain higher labor efficiency, and hence increased income, adding to precautionary savings that can be used to expand production investment, thereby promoting the household’s overall quality of life; under the “stabilizing effect”, better health of the family members reduce the long-term medical expenses caused by physical diseases, adding certainty to future access to income and thus effectively lowering the probability of poverty [85,86,87].

Additionally, the ratio of medical out-of-pocket expenditure to income will directly affect the medical service demand and health and well-being of the insured. The research results show that the higher the proportion of out-of-pocket expenditure in the income, the higher the VEP. This finding supports the Hypothesis 3 of this study. Many analyses show that poor people are more susceptible to medical and health expenditure, and the lower the income, the more exposed to disease risks [88,89]. The greater the burden of medical expenditure on the family, the more likely it is to fall into poverty [90]. When health is affected, the heightened medical burden will squeeze non-medical consumption, affecting the financial ability of poor families. A vicious circle may form when poverty and disease interact as both cause and effect. Since poor families are more exposed to the risk of poverty and disease, breaking them free from this vicious circle will be of great significance for effectively reducing their VP [91].

In addition, the increase in the number of chronically ill or the number of critically ill patients in the rural registered household increased its VEP, significantly tilting the probability balance towards the poverty side. This finding supports Hypotheses 4 and 5 in this study. Generally, the decline in income and the increase in medical expenses caused by the limitation of labor capacity after falling ill lift the probability of poverty to a certain extent [48]. The more ill members in the household, the more exposure it has to health shocks, and the more likely it is that it will fall into poverty in future.

In order to better illustrate the effect of medical reimbursement on the poverty risk brought about by the higher number of patients in rural households, this study multiplied the inpatient reimbursement rate with the number of the chronically ill and the number of the critically ill, respectively, multiplied the overall reimbursement rate with the number of the chronically ill and the number of the critically ill, respectively, and used the logit model to verify. The results found that the increase in the overall reimbursement rate and the inpatient reimbursement rate could partially offset the poverty risk caused by the higher number of the chronically ill. Chronic diseases are characterized by a long course of disease, which leads to a continuous health impact. By increasing the medical burden on patients, chronic diseases affect their investment in human capital and productivity, leading to a continuous decline in future income and in the ability to resist poverty risk [39,41,92]. Medical insurance changes the price faced by patients through the design of differentiated reimbursement rate and guides their health-seeking behavior [93]. Therefore, by increasing the overall reimbursement rate and the inpatient reimbursement rate, it is possible to reduce the medical expenses of poor families and stimulate their activity in seeking health, thereby improving their health and their resilience against poverty risks. On the contrary, in this study, the poverty risk arising from the higher number of the critically ill in the family increased with the increase in the overall reimbursement rate and the inpatient reimbursement rate. A possible explanation for this result is that, compared with chronic diseases, critical diseases pose a serious threat to the health status of patients, with higher costs, precise curative effects and longer duration. Under the circumstances of rising reimbursement rates and effective medical security, poor households will choose more active treatment and increase the activity of seeking medical treatment in the face of critical illness. Moreover, because people living in rural areas may have limited access to medical resources, they are more inclined to choose a higher-level hospital or a hospital outside the province rather than a community (town-level) health service institution for treatment after falling critically ill [94]. The transportation and accommodation expenses that may be incurred for medical treatment in a different place will increase other economic expenditures besides medical expenses [95]. Therefore, in the short term, the increase in the overall reimbursement rate will lead to more visits to hospitals, increased expenditure and missed work [43,96]. The increase in medical expenses due to disease and the cost of missed work due to the decline in physical quality will subject economically disadvantaged social groups to higher direct and indirect economic burdens, reduce their ability to withstand disease risks, and increase the probability of poverty [32,33].

Objectively, there are still some deficiencies in this study, which need to be further addressed in follow-up research. First of all, this study did not explore the differences among the registered poor households in rural areas. Future research can explore whether the TMS policy has different effects on poor households registered in different rural areas, so as to support research on improving the stratified and classified social assistance system [97]. Secondly, in addition to the medical reimbursement rate, there may be synergistic effects of other health poverty alleviation policies, such as family doctor contract services, medical assistance policies, etc. [71]. Finally, the COVID-19 pandemic increases the probability of rural households falling into poverty due to health losses, economic shutdowns, mass unemployment and the implementation of social isolation measures [98]. Our analysis failed to look deeply due to data limitations. In the follow-up research, the first-hand survey data can be used to further explore the pandemic’s action on the VP of rural low-income families [9].

## 6. Conclusions

In general, this study reveals the effect of the TMS policy on the VEP of Chinese rural households by analyzing the pooled cross-sectional dataset of registered poor households in Yunnan province, China. The research results show that increases in the overall reimbursement rate or the reimbursement rate for critical illness treatment effectively reduced the VEP; decreases in the proportion of out-of-pocket medical expenses to income effectively reduced the VEP; increases in the number of chronically ill members in the family increased its VEP, which could be offset by increasing the overall reimbursement rate or the inpatient reimbursement rate; increases in the number of critically ill members in the family increased its VEP, which, however, would increase in the short term with the increase in the rate of overall reimbursement rate or the rate of inpatient reimbursement.

Although there are certain limitations, the discussion of the TMS policy and the VEP of registered poor households in Yunnan province, China has important contemporary value and academic significance. Since the implementation of poverty alleviation through healthcare in 2020, China has scored great progress in building its medical security system. The TMS system takes basic medical security as the main body, critical illness insurance as a supplement and medical assistance as the foundation to provide comprehensive health protection for the rural poor, providing robust health support for the overall victory of poverty alleviation [99]. However, the total elimination of absolute poverty is not tantamount to the disappearance of the poverty threat posed by disease. At present, China is in the transitional stage of consolidating the achievements of poverty alleviation and effectively dovetailing with rural revitalization. The outbreak of the COVID-19 pandemic in late December 2019 further highlighted the importance of TMS for rural low-income families. Combined with the results of data analysis, the following suggestions are put forward. On the one hand, scientific methods should be adopted to optimize the medical reimbursement rate design with the health status and payment ability of rural low-income families taken into account fully, and more medical insurance assistance should be given to rural low-income families with critically ill patients [43]. On the other hand, it is necessary to diversify the social and economic scenes in the countryside, enhance the income-generating capacity of rural low-income families according to the actual local conditions, and reduce the out-of-pocket medical expenses as a proportion to total income [86]. In addition, it is recommended to widely carry out publicity and education on basic knowledge and skills of health literacy among low-income populations, strengthen the publicity of prevention and treatment knowledge for chronic disease and critical disease in low-income areas, and improve the health awareness of low-income populations [100].

## Figures and Tables

**Table 1:** Variables.

Category	Variable		Definition
Explained variable	Probability of poverty 1		Whether to have the probability of poverty when VEP exceeds 29% (1 = Yes, 0 = No)
	Probability of poverty 2		Whether to have the probability of poverty when VEP exceeds 50% (1 = Yes, 0 = No)
Explanatory variables	Inpatient reimbursement rate		Annual compensation rate of inpatient expenses for the registered poor household under relatively serious health shocks in the designated hospital during (%)
	Overall reimbursement rate		Annual compensation rate of medical expenses for all medical services including inpatient and outpatient treatment of the registered poor household in the designated hospital (%)
	Ratio of out-of-pocket expenses to income		Out-of-pocket medical expenses as a proportion to annual total income of the registered poor household in the current year (%)
	Intersection term between reimbursement rate and number of patients in the family		Inpatient reimbursement rate × number of the chronically ill
			Inpatient reimbursement rate × number of the critically ill
			Overall reimbursement rate × number of the chronically ill
			Overall reimbursement rate × number of the critically ill
Control variables	Household population	Number of migrant workers	Number of migrant workers in the family
		Number of the chronically ill	Number of the chronically ill in the family
		Number of the critically ill	Number of the critically ill in the family
	Characteristics of the household head	Education level of the household head	Highest educational attainment of the household head (1 = no education, 2 = preschool, 3 = primary school, 4 = first grade of junior high school, 5 = second grade of junior high school, 6 = third grade of junior high school, 7 = first grade of senior high school, 8 = second grade of senior high school, 9 = third grade of senior high school, 10 = first grade of secondary vocational school, 11 = second grade of secondary vocational school, 12 = third grade of secondary vocational school, 13 = first grade of higher vocational school, 14 = second grade of higher vocational school, 15 = third grade of higher vocational school, 16 = first grade of technical school, 17 = second grade of technical school, 18 = third grade of technical school, 19 = fourth grade of technical school, 20 = freshman, 21 = sophomore, 22 = junior, 23 = senior, 24 = fifth grade of higher education, 25 = graduate and above)
		Age	Age of the head of the household
	Agricultural management status	Cultivated area	Cultivated area (mu, 1 square kilometer = 1500 mu)
		Forest area	Forest area (mu)
		Whether to have joined a farmers’ specialized cooperative	Whether to be a member of a specialized agricultural cooperative (1 = Yes, 0 = No)
	Location conditions	Whether radio and TV are available	Whether radio and TV channels are available (1 = Yes, 0 = No)
		Distance from rural main roads	Distance from rural main roads (km)
		Whether there are hardened roads connected to the township	Whether there are asphalt/concrete roads that lead to other villages/towns (1 = Yes, 0 = No)
		Whether there are passenger shuttle buses	Whether shuttle buses are available (1 = Yes, 0 = No)
	Village-level economic characteristics	Village-level collective economic income	Unit: CNY 10,000
		Number of farmers’ specialized cooperatives	Unit: Individual

**Table 2:** Descriptive Statistics of Variables.

Variables	N	Mean	Sd	Min	P50	Max
Explained variable						
Probability of poverty 1	1,448,496	0.24	0.42	0.00	0.00	1.00
Probability of poverty 2	1,448,496	0.12	0.32	0.00	0.00	1.00
Medical reimbursement						
Inpatient reimbursement rate	1,448,138	79.03	15.28	0.00	90.00	100.00
Overall reimbursement rate	1,448,496	75.30	13.86	0.04	78.89	99.99
Out-of-pocket expenses as a percentage of income	1,448,496	13.66	20.91	0.02	6.63	190.79
Number of the sick						
Number of the chronically ill	1,448,496	0.41	0.65	0.00	0.00	4.00
Number of the critically ill	1,448,496	0.12	0.35	0.00	0.00	3.00
Characteristics of the household head						
Education level	1,448,496	2.13	0.62	1.00	2.00	6.00
Age	1,448,496	50.82	12.57	−1.00	50.00	98.00
Agricultural management status						
Cultivated area	1,448,496	6.83	8.96	0.00	4.00	316.40
Forest area	1,448,496	13.71	39.45	0.00	3.00	1200.00
Whether to join the farmers’ specialized cooperative	1,448,496	0.43	0.50	0.00	0.00	1.00
Location conditions						
Whether radio and television are available	1,448,496	0.96	0.20	0.00	1.00	1.00
Distance from rural main roads	1,448,496	1.23	4.25	0.00	0.30	2000.00
Whether there are hardened roads leading to the township	1,448,496	0.96	0.19	0.00	1.00	1.00
Whether there are passenger shuttle buses	1,448,496	0.75	0.43	0.00	1.00	1.00
Village-level economic characteristics						
Village-level collective economic income	1,448,496	0.07	1.87	0.00	0.03	206.00
Number of farmers’ specialized cooperatives	1,448,496	3.16	4.39	0.00	2.00	267.00

**Table 3:** Model Estimation Results.

	Inpatient Reimbursement Rate		Overall Reimbursement Rate	
	Coefficient	Odds Ratio	Coefficient	Odds Ratio
Inpatient reimbursement rate	−0.042 ***	0.959 ***		
	(0.000)	(0.000)		
Inpatient reimbursement rate × number of the chronically ill	−0.003 ***	0.997 ***		
	(0.000)	(0.000)		
Inpatient reimbursement rate × number of the critically ill	0.001 **	1.001 **		
	(0.000)	(0.000)		
Overall reimbursement rate			−0.045 ***	0.956 ***
			(0.000)	(0.000)
Overall reimbursement rate × number of the chronically ill			−0.004 ***	0.996 ***
			(0.000)	(0.000)
Overall reimbursement rate × number of the critically ill			0.002 ***	1.002 ***
			(0.001)	(0.001)
Number of the chronically ill	0.226 ***	1.254 ***	0.234 ***	1.263 ***
	(0.020)	(0.025)	(0.021)	(0.027)
Number of the critically ill	0.156 ***	1.169 ***	0.114 ***	1.120 ***
	(0.035)	(0.040)	(0.037)	(0.041)
Out-of-pocket expenses as a percentage of income	0.004 ***	1.004 ***	0.006 ***	1.006 ***
	(0.000)	(0.000)	(0.000)	(0.000)
Number of migrant workers	−1.405 ***	0.245 ***	−1.432 ***	0.239 ***
	(0.008)	(0.002)	(0.008)	(0.002)
Household head’s education	−0.256 ***	0.774 ***	−0.256 ***	0.774 ***
	(0.004)	(0.003)	(0.004)	(0.003)
Household head’s age	−0.012 ***	0.988 ***	−0.012 ***	0.988 ***
	(0.000)	(0.000)	(0.000)	(0.000)
Cultivated area	−0.004 ***	0.996 ***	−0.003 ***	0.997 ***
	(0.000)	(0.000)	(0.000)	(0.000)
Forest area	−0.005 ***	0.995 ***	−0.005 ***	0.995 ***
	(0.000)	(0.000)	(0.000)	(0.000)
Whether to have joined a farmers’ specialized cooperative	−1.771 ***	0.170 ***	−1.787 ***	0.168 ***
	(0.007)	(0.001)	(0.007)	(0.001)
Whether to have access to radio and television	−1.933 ***	0.145 ***	−1.937 ***	0.144 ***
	(0.011)	(0.002)	(0.011)	(0.002)
Distance from rural main roads	0.018 ***	1.018 ***	0.017 ***	1.017 ***
	(0.001)	(0.001)	(0.001)	(0.001)
Where there are hardened roads leading to the township	−1.593 ***	0.203 ***	−1.607 ***	0.201 ***
	(0.012)	(0.002)	(0.012)	(0.002)
Whether there are passenger shuttle buses	−0.315 ***	0.730 ***	−0.317 ***	0.728 ***
	(0.005)	(0.004)	(0.005)	(0.004)
Village-level collective economic income	0.018 ***	1.018 ***	0.018 ***	1.018 ***
	(0.001)	(0.001)	(0.001)	(0.001)
Number of farmers’ specialized cooperatives	−0.052 ***	0.950 ***	−0.055 ***	0.947 ***
	(0.001)	(0.001)	(0.001)	(0.001)
_cons	7.498 ***		7.589 ***	
	(0.027)		(0.028)	
N	1,448,138	1,448,138	448,496	1,448,496
Log likelihood (LL)	−507,572.64	−507,572.64	−510,255.2	−510,255.2
Pseudo *R*^2^	0.357	0.357	0.354	0.354

Note: ** denotes that the variables are significant at the 5% confidence level; *** denotes that the variables are significant at the 1% confidence level. Robust standard deviations are in parentheses.

**Table 4:** Robustness Test.

	Inpatient Reimbursement Rate		Overall Reimbursement Rate	
	Coefficient	Odds Ratio	Coefficient	Odds Ratio
Inpatient reimbursement rate	−0.034 ***	0.966 ***		
	(0.000)	(0.000)		
Inpatient reimbursement rate × number of the chronically ill	−0.006 ***	0.994 ***		
	(0.000)	(0.000)		
Inpatient reimbursement rate × number of the critically ill	0.002 ***	1.002 ***		
	(0.001)	(0.001)		
Overall reimbursement rate			−0.039 ***	0.962 ***
			(0.000)	(0.000)
Overall reimbursement rate			−0.039 ***	0.962 ***
			(0.000)	(0.000)
Overall reimbursement rate × number of the chronically ill			−0.007 ***	0.993 ***
			(0.000)	(0.000)
Overall reimbursement rate × number of the critically ill			0.002 ***	1.002 ***
			(0.001)	(0.001)
Number of the chronically ill	0.246 ***	1.279 ***	0.291 ***	1.338 ***
	(0.022)	(0.028)	(0.025)	(0.033)
Number of the critically ill	0.356 ***	0.700 ***	−0.328 ***	0.721 ***
	(0.039)	(0.027)	(0.043)	(0.031)
Out-of-pocket expenses as a percentage of income	0.006 ***	1.006 ***	0.007 ***	1.007 ***
	(0.000)	(0.000)	(0.000)	(0.000)
Number of migrant workers	−1.648 ***	0.192 ***	−1.672 ***	0.188 ***
	(0.016)	(0.003)	(0.016)	(0.003)
Education level of the head of the household	−0.228 ***	0.796 ***	−0.231 ***	0.794 ***
	(0.005)	(0.004)	(0.005)	(0.004)
Age of the household head	−0.010 ***	0.990 ***	−0.009 ***	0.991 ***
	(0.000)	(0.000)	(0.000)	(0.000)
Cultivated area	−0.009 ***	0.991 ***	−0.008 ***	0.992 ***
	(0.000)	(0.000)	(0.000)	(0.000)
Forest area	−0.006 ***	0.994 ***	−0.007 ***	0.993 ***
	(0.000)	(0.000)	(0.000)	(0.000)
Whether to have joined a farmers’ specialized cooperative	−2.289 ***	0.101 ***	−2.297 ***	0.101 ***
	(0.014)	(0.001)	(0.014)	(0.001)
Whether to have access to radio and television	−1.113 ***	0.329 ***	−1.113 ***	0.329 ***
	(0.010)	(0.003)	(0.010)	(0.003)
Distance from rural main roads	0.035 ***	1.036 ***	0.034 ***	1.035 ***
	(0.001)	(0.001)	(0.001)	(0.001)
Where there are hardened roads leading to the township	−1.657 ***	0.191 ***	−1.663 ***	0.189 ***
	(0.010)	(0.002)	(0.010)	(0.002)
Whether there are passenger shuttle buses	−0.144 ***	0.866 ***	−0.144 ***	0.866 ***
	(0.006)	(0.006)	(0.006)	(0.006)
Village-level collective economic income	0.008 ***	1.008 ***	0.008 ***	1.008 ***
	(0.001)	(0.001)	(0.001)	(0.001)
Number of farmers’ specialized cooperatives	−0.068 ***	0.934 ***	−0.070 ***	0.932 ***
	(0.001)	(0.001)	(0.001)	(0.001)
_cons	4.978 ***		5.174 ***	
	(0.029)		(0.029)	
N	1,448,138	1,448,138	1,448,496	1,448,496
Log likelihood (LL)	−362,290.38	−362,290.38	−362,586.8	−362,586.8
Pseudo *R*^2^	0.312	0.312	0.312	0.312

Note: *** denotes that the variables are significant at the 1% confidence level. Robust standard deviations are in parentheses.

## Data Availability

The data are derived from Yunnan province’s database on registered poor households provided by the Yunnan Provincial Poverty Alleviation Office and medical reimbursement database provided by the Yunnan Provincial Medical Insurance Bureau. We have signed a confidentiality agreement with the two agencies, so data sharing is not applicable.
